# Monitoring Leiomyoma Response to Uterine Artery Embolization Using Diffusion and Perfusion Indices from Diffusion-Weighted Imaging

**DOI:** 10.1155/2017/3805073

**Published:** 2017-08-27

**Authors:** Mengqiu Cao, Lijun Qian, Xuebin Zhang, Xinjun Suo, Qing Lu, Huilin Zhao, Jialin Liu, Jianxun Qu, Yan Zhou, Jianrong Xu, Shiteng Suo

**Affiliations:** ^1^Department of Radiology, Renji Hospital, School of Medicine, Shanghai Jiao Tong University, Shanghai 200127, China; ^2^Department of Interventional Oncology, Renji Hospital, School of Medicine, Shanghai Jiao Tong University, Shanghai 200127, China; ^3^School of Medical Imaging, Tianjin Medical University, Tianjin 300070, China; ^4^School of Biomedical Engineering, Shanghai Jiao Tong University, Shanghai 200240, China; ^5^GE Healthcare China, Shanghai 201203, China

## Abstract

**Purpose:**

To investigate the potential of diffusion and perfusion indices (ADC and perfusion fraction *f*) from DWI at 3.0 T in monitoring treatment response to uterine artery embolization (UAE) at 6-month follow-up.

**Methods:**

Twelve female patients with uterine fibroids who underwent 3.0-T pelvic DWI before and 6 months after UAE were included. ADC and perfusion fraction *f* were calculated from DWI. The Wilcoxon signed-rank test and Spearman rank correlation test were used for statistics.

**Results:**

Seventeen fibroids were studied. The median ADCs showed a significant increase from 1.20 × 10^−3^ mm^2^/s (range, 0.86–1.66 × 10^−3^ mm^2^/s) at baseline to 1.56 × 10^−3^ mm^2^/s (range, 1.00–1.86 × 10^−3^ mm^2^/s) at 6-month follow-up (*P* = 0.0003). Conversely, the median perfusion fraction *f* was significantly decreased after UAE (*P* = 0.0001), with a median pre-UAE value of 14.2% (range, 6.7%–17.6%) and a median post-UAE value of 9.2% (range, 3.2%–14.6%). Significant correlations were found between fibroid volume reduction rate and percentage changes in ADC and perfusion fraction *f* at 6-month follow-up relative to baseline, with *ρ* values of −0.50 (*P* = 0.04) and 0.55 (*P* = 0.02), respectively.

**Conclusion:**

ADC and perfusion fraction *f* obtained from DWI at 3.0 T may help to evaluate treatment response to UAE.

## 1. Introduction

Uterine leiomyomas (fibroids) are the most common benign gynecological tumors, primarily affecting women of reproductive age [1]. The estimated cumulative incidence of fibroids by the age of 50 years is above 70% in the USA [2]. Women with symptomatic fibroids may present with menorrhagia, pelvic pain, and bulk-related symptoms such as bladder irritation or constipation with a substantial negative effect on their life quality [3, 4]. Uterine artery embolization (UAE) has been established as a minimally invasive, safe, and effective treatment for symptomatic fibroids, offering the advantages of potential preservation of uterus and fertility and avoidance of surgical procedures associated with higher risks and longer hospitalization [5, 6].

MRI has been demonstrated to be the most accurate imaging tool that is currently available to assess fibroid changes after UAE [7]. Typically, a volume reduction of fibroids is considered as successful morphologic imaging criterion. A previous study showed that poor clinical improvement was related to a lesser volume reduction rate after UAE [8]. In routine clinical practice, contrast-enhanced MRI is useful to assess the volume and vascularity of fibroids by contrast material bolus injection, thus aiding in treatment monitoring.

Diffusion-weighted imaging (DWI) is a contrast-free MRI technique that allows the mapping of the diffusion process of water molecules in human tissues. By measuring the diffusion-weighted signal attenuation at two or more *b*-values (a combination of diffusion gradient strength and pulse duration), quantitative data such as apparent diffusion coefficient (ADC) can be yielded. Several studies have shown that ADC may be a useful imaging marker to monitor the treatment response after UAE on 1.5-T MRI platforms [9–12]. However, the previous studies produced contradictory results, particularly in terms of whether post-UAE ADC increases or decreases compared with baseline measurement [9–12] (Table 1). Additionally, to our knowledge, no previous data have been published at 3.0-T MRI until now.

According to the intravoxel incoherent motion (IVIM) theory, the water microscopic motion in each image voxel includes not only pure thermally driven molecular diffusion but microperfusion of blood in the capillary network [13]. As a result, increasing *b*-values lead to a biexponential diffusion-weighted signal decay within each voxel. The percentage of total signal arising from the microvascular compartment is defined as perfusion fraction *f* in the IVIM mathematical expression [13]. With extensive IVIM studies in experimental and clinical settings, the perfusion fraction *f* has proved valuable for evaluating treatment response to antivascular therapies [14, 15]. We surmised that perfusion fraction *f* might be useful in monitoring the microvascular change change of fibroids after UAE, because UAE would directly induce devascularization and result in cell death and necrosis.

Therefore, the purpose of this study was to investigate the potential of diffusion and perfusion indices (ADC and perfusion fraction *f*) from DWI at 3.0 T in the quantitative assessment of the treatment response to UAE at 6-month follow-up.

## 2. Materials and Methods

### 2.1. Subjects

This study was approved by our institutional review board and the requirement for informed consent was waived. From February 2012 to December 2013, 12 consecutive patients (median age, 42 years; range, 24–56 years) with symptomatic fibroids who satisfied the following criteria were recruited in this retrospective study:

1. Symptomatic fibroids were treated with UAE.
2. UAE was performed by the same interventionist using the same embolization protocol.
3. Pelvic MRI including DWI and IVIM sequences was obtained before and 6 months after the embolization procedure.

### 2.2. MRI Protocol

All patients underwent pre- and post-UAE MRI examinations by using the same 3.0-T MRI system (HDxt; General Electric Medical Systems, Waukesha, WI, USA) equipped with a multichannel phased-array coil. Conventional anatomical imaging sequences included axial T2-weighted fast spin echo (repetition time [TR]/repetition time [TE], 3500 ms/112 ms; slice thickness, 5 mm; gap, 1 mm; field of view [FOV], 320 × 320 mm; and matrix, 320 × 256), coronal T2-weighted fast spin echo (TR/TE, 3400 ms/100 ms; slice thickness, 5 mm; gap, 1 mm; FOV, 300 × 300 mm; and matrix, 320 × 224), sagittal T2-weighted fast spin echo (TR/TE, 3600 ms/100 ms; slice thickness, 4 mm; gap, 1 mm; FOV, 280 × 280 mm; and matrix, 320 × 224), and axial T1-weighted spin echo (TR/TE, 660 ms/7 ms; slice thickness, 5 mm; gap, 1 mm; FOV, 320 × 320 mm; and matrix, 352 × 192).

Diffusion-weighted MR images were obtained in the axial plane using a single-shot echo-planar imaging sequence (TR/TE, 5200 ms/67 ms; slice thickness, 4 mm; gap, 1 mm; FOV, 320 × 320 mm; matrix, 96 × 130; number of averages, 1–4; and *b*-values, 0 and 1000 s/mm^2^ for ADC calculation, and 0, 50, 100, 200, 500, 800, 1000, and 1200 s/mm^2^ for IVIM analysis). Isotropic DWI was generated using the three orthogonal-axis images. Contrast-enhanced T1-weighted imaging was carried out using the three-dimensional liver acquisition with volume acquisition (LAVA) sequence following injection of 0.1 mmol/kg gadolinium (Magnevist, Bayer Schering Pharma AG, Germany); the sequence parameters were as follows: TR/TE, 3 ms/1 ms; FOV, 340 × 340 mm; matrix, 256 × 224; and flip angle, 11°.

### 2.3. Image Analysis

Fibroids with a minimum diameter of 2 cm in at least one dimension on pre-UAE multiplanar T2-weighted images were chosen for image analysis [16]. One to three fibroids were studied in each patient [11]. If there were more than three fibroids identified, only the largest three fibroids were included. Each fibroid was segmented on postcontrast images using open source software ITK SNAP 2.4 (http://www.itksnap.org) [17], and the three-dimensional volume was calculated as a product of the delineated areas on all slices and the slice thickness [18]. For each fibroid, volume reduction rate (VRR) between baseline (Vol^pre^) and 6-month follow-up (Vol^post^) was calculated by using the following equation: VRR = (Vol^pre^ − Vol^post^)/Vol^pre^ · 100%.

Diffusion-weighted images were postprocessed by using an in-house software program implemented in MATLAB (release 2014b; MathWorks, Natick, MA, USA), to generate the parametric maps of ADC and IVIM-derived perfusion fraction *f* on a voxelwise basis. The ADC was calculated with a monoexponential model including *b*-values of 0 and 1000 s/mm^2^: *S*(*b*) = *S*_0_ · exp⁡(−*b*ADC), where *S*(*b*) and *S*_0_ denote the diffusion-weighted signal intensities obtained with *b*-values of 1000 s/mm^2^ and 0, respectively. Furthermore, the multi-*b*-value diffusion-weighted images were analyzed using the biexponential IVIM model: *S*(*b*) = *S*_0_ · [*f* · exp⁡(−*bD*^*∗*^) + (1 − *f*) · exp⁡(−*bD*)], where *D* and *D*^*∗*^ are the diffusion coefficient and pseudodiffusion coefficient related to the tissue diffusivity and microvascular perfusion, respectively. To obtain a more robust estimate for the perfusion fraction *f*, a segmented fitting approach was applied as previously published [19, 20]. This approach takes advantage of the fact that *D*^*∗*^ is significantly larger than *D* and its influence on diffusion-weighted signal can be neglected when *b*-value > 200 s/mm^2^. Thus, in this high *b*-value regime, *D* can be determined by linearly fitting the natural log of monoexponential equation described above. Keeping the calculated *D* value fixed, *D*^*∗*^ and *f* were nonlinearly fitted subsequently using the entire *b*-value distribution. All curve-fitting analyses were performed in MATLAB using a Levenberg-Marquardt routine. Oval regions of interest (ROIs) were manually drawn by two authors in consensus (M. C. and L. Q., with 4 years and 10 years of experience in interpretation of gynecological MRI, resp.) on DWI, and the ROIs were then copied to the corresponding ADC and *f* maps to obtain the quantitative values. Percentage changes in ADC and *f* after UAE relative to baseline were calculated as follows: ΔValue = (Value^post^ − Value^pre^)/Value^pre^ · 100%.

In addition, a ROI was measured in air on the diffusion-weighted image with maximal *b*-value (1200 s/mm^2^). Signal-to-noise ratio (SNR) was calculated as the ratio between the mean signal intensity of the fibroid and the standard deviation of air.

### 2.4. UAE Procedure

UAE was performed by an interventional radiologist (X. Z., with 19 years of experience in interventional therapy). Unilateral right femoral artery access and Waltman loop technique were used in all cases. A 4.0-Fr Cobra catheter (Cook, Bloomington, IN, USA) was placed in the uterine artery, and a coaxial 2.6-Fr microcatheter (Stride; Intecc Co., Ltd., Japan) was advanced distally into the ascending branch of uterine artery. The primary embolic agent was nonspherical polyvinyl alcohol particles (Alicon Medical Co., Ltd., Hangzhou, China) of size 250–560 *μ*m mixed with 100 mL of 1 : 1 saline solution : contrast agent mixture. Embolization was finished until stasis was achieved in the uterine artery. Fentanyl Transdermal System patch (2.5 milligrams) (Durogesic, Changzhou, China) was administered the day before UAE for management of pain during and after UAE.

### 2.5. Statistical Analysis

Data are presented as median (range). Mann–Whitney *U* test was used to compare ADC and perfusion fraction *f* between young (<45 years) and old (≥45 years) subject groups. To determine whether there were differences in DWI parameter values (ADC and perfusion fraction *f*) before and 6 months after UAE, the Wilcoxon signed-rank test was used. Box-whisker plots were obtained to illustrate the differences. To assess the relationships between percentage changes in ADC/*f* after UAE and VRR, the Spearman rank correlation test was performed. *P* values < 0.05 were considered statistically significant. All analyses were performed using GraphPad Prism 5 (GraphPad Software Inc., San Diego, CA, USA).

## 3. Results

A total of 17 fibroids in 12 patients were studied. The majority of patients (9/12, 75.0%) had one fibroid, while 16.7% (2/12) had 2 fibroids, and 8.3% (1/12) had 3 fibroids. The median time interval between pre-UAE MRI and UAE was 5 days (range, 1–8 days), and the post-UAE MRI was conducted approximately 6 months following UAE. UAE was successfully carried out in all 12 patients with no major complications detected. All the patients reported symptomatic improvement at 6-month follow-up. Based on follow-up postcontrast imaging, 17 fibroids (100%) were categorized as having complete infarction.

The median volume of all fibroids was 67.9 cm^3^ (range, 7.3–657.9 cm^3^) before UAE and 21.5 cm^3^ (range, 1.0–223.7 cm^3^) at 6-month follow-up. The volume reduction in fibroids was significant at 6-month follow-up (*P* < 0.0001). The median VRR in fibroids was 54.8% (range, 25.8%–94.9%) on the 6-month follow-up MRI.

**Figure 1:**
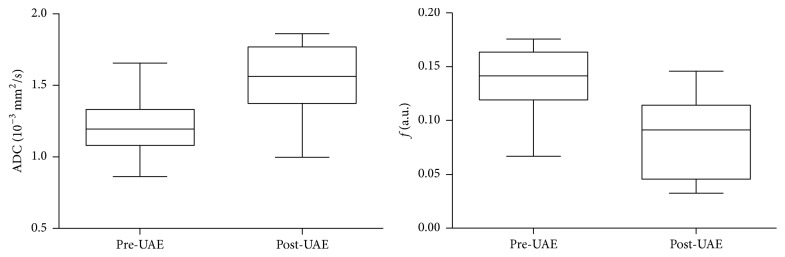
Box-whisker plots show ADC and perfusion fraction *f* before and 6 months after UAE. Significant differences were found between pre- and post-UAE ADCs (*P* = 0.0003) and *f* values (*P* = 0.0001) by using the Wilcoxon signed-rank test. Lines in boxes indicate medians and boundaries of boxes indicate lower and upper quartiles. Whiskers display the maximum and minimum.

**Figure 2:**
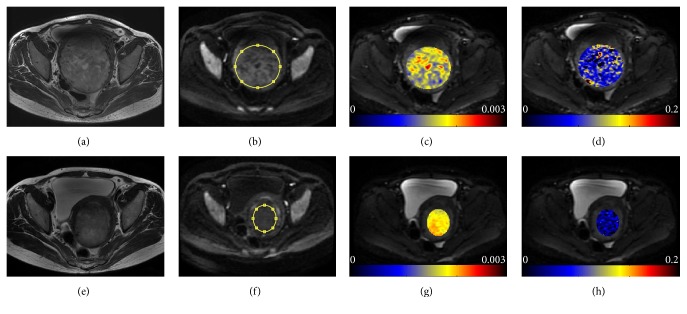
Images obtained in a 42-year-old woman treated with UAE for symptomatic fibroid before (a–d) and 6 months after embolization (e–h). (a, e) T2-weighted images demonstrate a submucosal fibroid which is oval shaped and heterogeneously hypointense. At 6-month follow-up, the fibroid exhibits remarkable reduction in size. (b, f) Placement of oval ROIs within the lesion on DWI. (c, g) ADC maps in the ROIs show increased diffusion properties at 6-month follow-up relative to baseline (1.85 × 10^−3 ^mm^2^/s versus 1.61 × 10^−3 ^mm^2^/s). (d, h) IVIM-derived perfusion fraction *f* maps in the ROIs show decreased microvascular perfusion/blood flow at 6-month follow-up relative to baseline (4.13% versus 7.98%).

Median SNR on the diffusion-weighted image with *b*-value of 1200 s/mm^2^ was 42.9 (range, 12.8–80.3) for pre-UAE fibroids and 22.0 (range, 9.9–40.9) for post-UAE fibroids. No significant differences in pre-UAE ADC and perfusion fraction *f* were observed between the young and old groups (*P* = 0.23 and 0.28, resp.). The median values of the percentage change in post-UAE ADC and perfusion fraction *f* relative to baseline are summarized in Table 2 and represented in respective box-whisker plots (Figure 1). The median ADCs showed a statistically significant increase from 1.20 × 10^−3 ^mm^2^/s (range, 0.86–1.66 × 10^−3 ^mm^2^/s) at baseline to 1.56 × 10^−3 ^mm^2^/s (range, 1.00–1.86 × 10^−3 ^mm^2^/s) at 6-month follow-up (*P* = 0.0003). Conversely, the median perfusion fraction *f* was significantly decreased after UAE (*P* = 0.0001), with a median pre-UAE value of 14.2% (range, 6.7%–17.6%) and a median post-UAE value of 9.2% (range, 3.2%–14.6%).  Figure 2 shows a representative case of a 42-year-old patient treated with UAE, indicating a decreased fibroid volume, an increased ADC value, and a decreased *f* value at 6-month follow-up.

**Figure 3:**
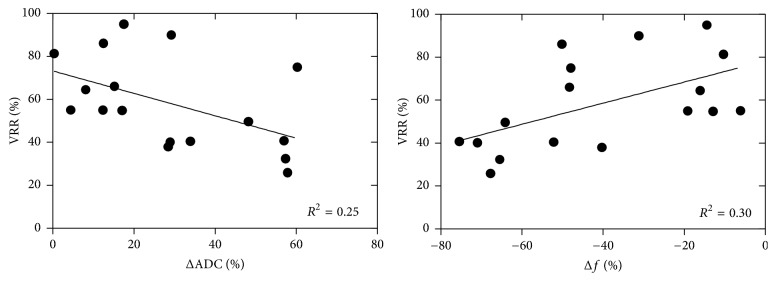
Graphs show relationships between fibroid volume reduction rate and percentage changes in ADC and perfusion fraction *f* at 6-month follow-up relative to baseline. The Spearman rank correlation test revealed significant correlations, with *ρ* values of −0.50 (*P* = 0.04) and 0.55 (*P* = 0.02), respectively.

The correlations between changes in ADC/*f* after UAE and VRR were evaluated to determine if the DWI parameters could predict fibroid volume response after UAE (Figure 3). The Spearman rank correlation test showed that ΔADC was negatively correlated with VRR (Spearman *ρ* = −0.50, *P* = 0.04) and Δ*f* was positively correlated with VRR (Spearman *ρ* = 0.55, *P* = 0.02), which indicated that a greater increase in ADC or a greater decrease in *f* would predict a smaller decrease in fibroid volume at 6-month follow-up.

## 4. Discussion

In this study, we performed 3.0-T DWI on patients before and 6 months after UAE. Monoexponential and biexponential IVIM analysis for diffusion-weighted images were successfully conducted on each subject. Detailed analysis of the acquired data demonstrates the feasibility of diffusion and perfusion indices obtained from DWI in monitoring fibroid response after UAE. Post-UAE ADC of fibroids was significantly higher than that measured at baseline, while IVIM-derived perfusion fraction *f* was significantly lower compared to pre-UAE measurement. Furthermore, percentage changes in ADC/*f* after UAE were significantly correlated with VRR (negative correlation for ADC and positive correlation for *f*), suggesting their predictive value for fibroid volume response at 6 months after UAE.

DWI (together with quantitative ADC map) is a rapidly evolving technique with potential to characterize tissue cellularity and cell membrane integrity [21]. In our study, a significant increase in ADC value occurred in fibroids treated with UAE, which is in agreement with the result of a recent study [12]. Kirpalani et al. showed that the pre- and post-UAE ADCs at 1.5 T were 1.30 and 1.68 × 10^-3 ^mm^2^/s at 6-month follow-up and they were significantly different (*P* < 0.0001) [12]. This presumably reflects a loss of cellularity, membrane integrity, and extracellular space expansion resulting from cell necrosis after embolization. Additionally, probably at least in part because of the accumulated clusters of uniform smooth muscle cells with collagen deposition [9], fibroids before embolization represent a more restricted diffusion pattern. However, three other earlier studies reported distinct results [9–11]. Liapi et al. evaluated 32 fibroids in 11 patients treated with UAE and found a significant drop in post-UAE ADC value across a follow-up interval of 99–239 days [9]. Ananthakrishnan et al. also showed that post-UAE ADC measured from 15 patients was lower than pre-UAE ADC at 6-month follow-up [10]. Faye et al. further studied the ADC changes in 27 fibroids in 17 patients at 1-week and 6-month follow-ups and reported a continued ADC decrease relative to pre-UAE measurement [11]. Liapi et al. and Ananthakrishnan et al. proposed that the drop in ADC following UAE might depend on the acute microscopic changes like dehydration [9, 10]. However, it should be emphasized that there may be progressive liquefaction of fibroids with increasing time between UAE and follow-up MRI [7]. Therefore, the change in post-UAE ADC is complex according to the pathophysiological state of fibroids. At present, the pathophysiology of fibroid infarction and its exact temporal evolution after embolization still remain incompletely understood and need further elucidation [22]. In addition, several other factors including patient population, imaging setting, and follow-up scheme may also account for the inconsistency of results in these studies.

As pointed out in the limitation of the previous studies [10–12], the simplified monoexponential algorithm for ADC calculation lacks accuracy in depicting the tissue characteristics of fibroids because the diffusion-weighted signal combines both diffusion and perfusion effects according to the IVIM theory [13]. In human tissues, diffusion-weighted data obtained with low *b*-values (<200 s/mm^2^) are more sensitive to perfusion effect, whereas data obtained with high *b*-values (>200 s/mm^2^) predominantly account for diffusion effect [23]. Specifically, these two components may have opposite effects on the diffusion-weighted signal attenuation of post-UAE fibroids: loss of cellularity (diffusion component) may increase ADC while devascularization (perfusion component) may decrease ADC. In this study, we for the first time applied IVIM diffusion-weighted MRI with a wide range of *b*-values (including low [<200 s/mm^2^]) to separate these two components and assess the perfusion changes of fibroids before and 6 months after UAE. The preliminary results showed a substantial drop in post-UAE measurement of perfusion fraction *f*. The perfusion changes after UAE revealed by IVIM can be explained by histologic changes. Because the perfusion fraction *f* is considered to represent blood volume [24], low vascularity would lead to deceased *f* value. The inherent vascular pattern of fibroids appears to represent a localized expansion of the myometrial vasculature [18]. Embolization procedure induces sluggish blood flow in myometrial vessels which would result in transient uterine ischemia [25]. Fibroids are prone to undergo devascularization as they seem to be more susceptible to ischemia, thereby representing reduced perfusion in tumors. DeSouza and Williams showed by dynamic contrast-enhanced MRI that fibroid perfusion was suppressed to baseline levels at 1-month and 4-month follow-ups [26]. In this study, all fibroids were categorized as having complete infarction on the 6-month follow-up MRI, which was consistent with the IVIM findings. Additionally, several studies assessing cerebral infarction (comparable with UAE as there is an acute vascular occlusion) using IVIM diffusion-weighted MRI also found a drop in perfusion fraction *f* in the ischemic lesion [27, 28], in agreement with the current results on UAE.

Nowadays, 3.0-T MRI systems are widely used in clinical practice. 3.0-T DWI is superior to 1.5 T in terms of higher SNR and greater spatial resolution. To our knowledge, this study represents the first human study of monitoring fibroid response to UAE using DWI at 3.0 T. Theoretically, the ADC and perfusion fraction *f* values are assumed to be independent of magnetic field strength. However, previous studies reported substantial variability in ADC and perfusion fraction *f* values at 1.5- and 3.0-T MRI systems [29–32], which have implications for the use of DWI in assessing diffusion and perfusion indices of various anatomic abnormalities including fibroids.

Our study have limitations. First, the sample size was relatively small, which may limit the statistical power. Large-scale prospective studies are merited to confirm and extend the present observations. Second, no pathological findings were available as none of the patients received myomectomy or hysterectomy in case of symptom worsening or tumor recurrence at 6-month follow-up. The underlying mechanism in diffusion-weighted parameter alterations after UAE still remains incompletely understood. Therefore, radiological-pathological correlation was merely hypothesized. Third, as the main purpose of the preliminary study was to investigate the role of diffusion and perfusion indices obtained from DWI in monitoring fibroid response after UAE, other imaging features derived from conventional sequences including T2-weighted, T1-weighted, and contrast-enhanced MRI were not taken into account. Although all patients showed significantly elevated ADC values and decreased perfusion fraction *f* values 6 months after UAE, there still remained an overlap between pre- and post-UAE quantitative measurements. This is probably due to the individual difference of patients, and DWI could only depict the diffusion-related features of fibroids. Utility of multiparametric MRI would certainly be worthwhile to explore in future studies.

## 5. Conclusion

In conclusion, diffusion and perfusion indices (ADC and perfusion fraction *f*) from DWI at 3.0 T may help to evaluate treatment response to UAE. They hold the potential to provide insight into physiological changes in fibroids after embolization, though further validation is needed in clinical practice.

## Figures and Tables

**Table 1:** Literature review of published pre- and post-UAE ADC values at 6-month follow-up.

Author, year	Magnetic field strength (T)	*b*-values (s/mm^2^)	Pre-UAE ADC (10^−3 ^mm^2^/s)	Post-UAE ADC (10^−3 ^mm^2^/s)
Liapi et al., 2005 [9]	1.5	0 and 500	1.74	1.22
Ananthakrishnan et al., 2012 [10]	1.5	0 and 1000	1.01 ± 0.39	0.48 ± 0.26
Faye et al., 2013 [11]	1.5	0 and 500	1.61	1.27
Kirpalani et al., 2014 [12]	1.5	0, 250, 500, and 750	1.30 ± 0.20	1.68 ± 0.24

**Table 2:** Measurements of ADC and perfusion fraction *f* at baseline and 6-month follow-up.

Parameter	Pre-UAE	Post-UAE	*P* value^*∗*^
ADC (10^−3 ^mm^2^/s)	1.20 (0.86–1.66)	1.56 (1.00–1.86)	0.0003
Perfusion fraction *f* (%)	14.2 (6.7–17.6)	9.2 (3.2–14.6)	0.0001

^*∗*^The Wilcoxon signed-rank test.
